# Efficacy and safety of Shulie'an capsules in treating chronic prostatitis: A multicenter, prospective, open-label clinical trial

**DOI:** 10.1515/jtim-2026-0078

**Published:** 2026-08-26

**Authors:** Yuzhuo Yang, Jing Peng, Xiansheng Zhang, Tao Jiang, Ning Zhang, Hui Jiang

**Affiliations:** Department of Urology, Peking University First Hospital; The Institution of Urology, Peking University; Beijing Key Laboratory of Urogenital Diseases (Male) Molecular Diagnosis and Treatment Center; National Urological Cancer Center, Beijing, China; Department of Urology, The First Affiliated Hospital of Anhui Medical University, Hefei, Anhui Province, China; Department of Andrology and Sexual Medicine, The Second Hospital of Dalian Medical University, Dalian, Liaoning Province, China; International Cancer Institute, Peking University Health Science Center, Beijing, China

**Keywords:** Shulie'an capsules, chronic prostatitis, efficacy, safety

## Abstract

**Background and Objectives:**

Chronic prostatitis is a highly prevalent urological disorder affecting millions of men globally and significantly impairing quality of life. The aim of this study was to evaluate the efficacy and safety of Shulie'an (SLA) capsules in patients with chronic prostatitis.

**Methods:**

In this multicenter, prospective, open-label, self-controlled clinical trial, 100 male patients with clinically confirmed chronic prostatitis were enrolled in China. The participants received oral SLA (two capsules per dose, three times daily) for 28 days. The primary endpoint was the change in the National Institutes of Health Chronic Prostatitis Symptom Index (NIH-CPSI) total score from baseline to endpoint. Secondary endpoints included NIH-CPSI individual domain scores and disappearance rate of traditional Chinese medicine (TCM) symptoms. Safety assessments involved monitoring and recording all adverse events throughout the study period.

**Results:**

After 28 days of treatment, SLA capsules exhibited significant clinical efficacy, with an overall response rate of 91.75%. The mean NIH-CPSI total score reduction was 9.2±5.82 and 15.0±6.79 points on the 14th and 28th day, respectively. Approximately 95.88% of the participants achieved more than 4-point reduction in the NIH-CPSI score from baseline after treating for 28 days. All symptom domains showed statistically significant improvements, with symptoms of pain being the most remarkable (complete resolution rate: 41.57%). Besides, after 28 days of treatment, the total TCM syndrome score of the participants significantly improved, and the treatment was considerably safe with no reported serious adverse events.

**Conclusion:**

This study demonstrated that SLA capsules are a promising therapeutic option for chronic prostatitis, significantly improving clinical symptoms and quality of life with a favorable safety profile.

## Introduction

Chronic prostatitis, a heterogeneous spectrum of inflammatory disorders targeting the prostate, is a prevalent urological condition in adult males, which accounts for approximately 8%–25% of urology outpatient visits worldwide.^[1,2]^ Epidemiological studies indicate that over 50% of affected men experience significant quality of life impairment, with reported incidence rates ranging from 6.0%–32.9% across different populations, which causes substantial patient suffering and imposes a considerable economic burden on healthcare systems.^[3, 4, 5]^

According to the National Institutes of Health (NIH) classification system, chronic prostatitis comprises four distinct categories: Type I (acute bacterial), Type II (chronic bacterial), Type III (chronic prostatitis/chronic pelvic pain syndrome, CP/CPPS), and Type IV (asymptomatic inflammatory).^[6,7]^ It has a remarkably heterogeneous clinical presentation and potentially involves infectious agents (including bacterial and mycoplasma infections), psychological comorbidities (such as depression and anxiety disorders), pelvic floor dysfunction, and autoimmune mechanisms. This multifactorial etiology contributes to the chronic, recalcitrant nature of the condition, imposing significant therapeutic challenges.^[6, 7, 8, 9]^ Levofloxacin (for bacterial forms) and terazosin (for voiding symptoms) are the current primary pharmacological management. However, clinical evidence reveals that levofloxacin has a limited efficacy in Type III prostatitis,^[10]^ whereas the utility of terazosin may be constrained by its hypotensive effects.^[11]^ These therapeutic limitations have spurred interest in exploring alternative treatment modalities, particularly traditional medicines.

The Shulie'an (SLA) capsule (National Medical Products Administration approval No. Z20025167) is a standardized traditional Chinese herbal preparation for chronic nonbacterial prostatitis with dampness-heat accumulation syndrome manifesting as lower urinary tract symptoms, including frequent urination, urgency, and dysuria. The primary active component, *Campylotropis trigonoclada* (Franch.) Schindl. (Leguminosae), known colloquially as “Daxue” or “Three-angled Vine,” has been ethnopharmacologically documented in Bai minority medicine for treating urological disorders, including prostatitis and nephrolithiasis.^[12, 13, 14, 15]^ Isoorientin is the core active component of *Campylotropis trigonoclada* (Franch.) Schindl. (Leguminosae).^[16]^ This compound reportedly exerts multiple pharmacological effects, including antioxidant, anti-inflammatory, neuroprotective, and antidepressant effects and metabolic regulatory activities.^[17,18]^ These effects are consistent with the key pathological processes involved in chronic prostatitis pathogenesis, such as inflammatory responses, neurogenic inflammation, oxidative stress, metabolic disorders, and depression-related comorbidities.^[19,20]^

Preclinical investigations have shown that SLA capsules exhibit dual modulatory effects on the urinary tract smooth muscle-contracting bladder detrusor, while relaxing the urethral strips in animal models. These pharmacological actions correlate with its traditional use in relieving symptoms of prostatitis. However, despite promising preclinical data, robust clinical evidence supporting its therapeutic applications remains scarce. To address this gap, we conducted a multicenter, open-label, self-controlled clinical trial to systematically evaluate the efficacy and safety profiles of SLA capsules in patients with chronic prostatitis. The aim of the study was to provide high-quality clinical data to inform therapeutic decision-making for this challenging condition.

## Methods

### Study approval

This multicenter clinical trial was conducted at three center institutions in China: Peking University First Hospital, The Second Hospital of Dalian Medical University, and The First Affiliated Hospital of Anhui Medical University. The study was performed in accordance with the Declaration of Helsinki and complied with Good Clinical Practice guidelines. The study protocol received approval from the Institutional Review Board and the Human Research Ethics Committee at each participating center prior to study initiation (Approval ID: KY2024–047–01; 2023yan548–002; PJ2024–01–28), registered with the Chinese Clinical Trial Registry (ChiCTR2500114151). All patients provided written informed consent before they were enrolled.

### Participants

**Figure 1:**
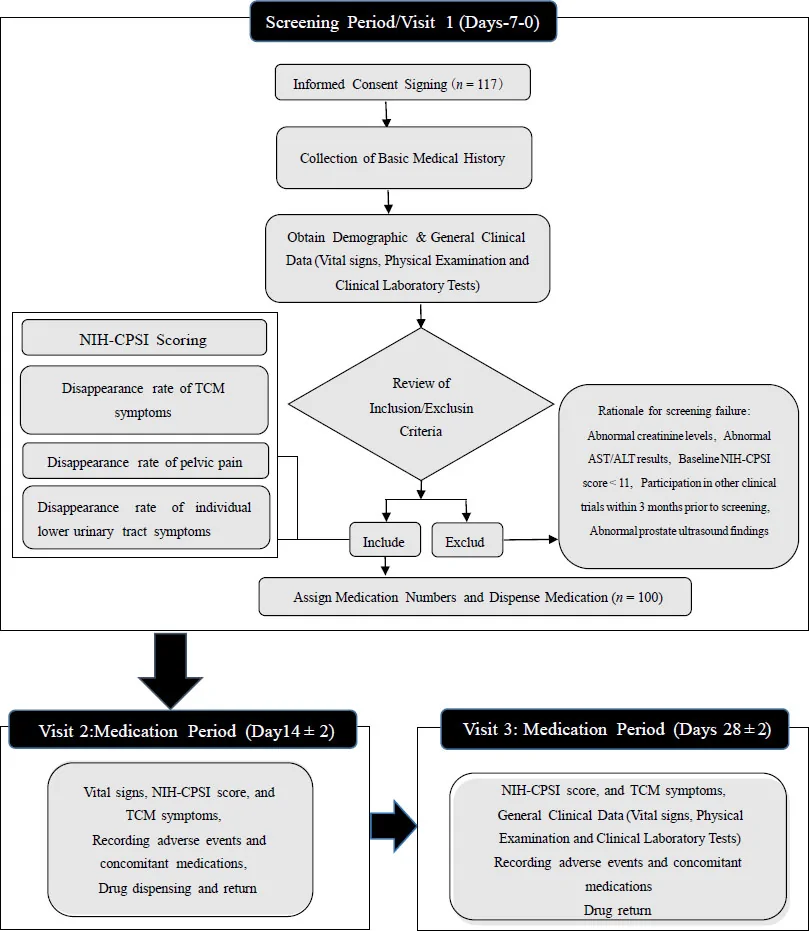
The flowchart of this clinical trial. The entire trial process comprises 3 visit time points. Among them, Day-7 to Day 0 is the baseline period, which includes the signing of patients' informed consent forms, assessment of inclusion and exclusion criteria, and collection of baseline clinical data. Day 14 and Day 28 are two follow-up visits, mainly involving the collection of data related to the NIH-CPSI scale, TCM symptoms, and adverse reactions. NIH-CPSI: National Institutes of Health Chronic Prostatitis Symptom Index; TCM: traditional Chinese medicine.

One hundred participants were enrolled in this clinical trial, and the study flowchart is shown in Figure 1. Eligible participants met the Western diagnostic criteria for chronic prostatitis, were aged between 18 and 50 years, had a baseline National Institutes of Health Chronic Prostatitis Symptom Index (NIH-CPSI) score of at least 11, experienced chronic prostatitis symptoms for ≥ 3 months, and voluntarily participated with informed consent. Patients with evidence of acute or chronic bacterial prostatitis associated with active infection, based on clinical presentation and routine laboratory examinations (including urinalysis), were excluded; therefore, the study population primarily consisted of patients with clinical features consistent with CP/CPPS. In addition, we excluded those with other conditions affecting evaluation (such as benign prostatic hyperplasia, prostate cancer, urinary tract infections, *etc*.), a history of prostate or bladder surgery, local pain from other lesions, severe primary diseases (involving heart, liver, kidney, *etc*.) or abnormal liver/kidney function, allergic constitution or known allergies to the trial drug. Exclusion also included the use of androgen inhibitors, antibiotics, *α*1-blockers, or other prostatitis treatments within the past 2 weeks, clear indications for surgery, family planning within 6 months, alcohol/drug abuse, participation in other clinical trials within 3 months, inability to cooperate (*e.g*., due to mental illness), or other conditions deemed unsuitable by the investigators. These criteria were designed to ensure precise target population selection, safety, reliable efficacy assessment, and ethical compliance.

### Study design

This was a multi-center, open-label, self-controlled clinical trial conducted to evaluate the efficacy and safety of SLA capsules in the treatment of chronic prostatitis. We systematically collected the subjective symptoms, objective signs, new-onset diseases, laboratory test results, and medical interventions were obtained from the patients. The primary outcome measure was the change in the NIH-CPSI score after 28 days of treatment from baseline. The NIH-CPSI scale, which ranges from 0 (best) to 43 (worst), comprises four subscales that are used to assess pain, urinary symptoms, symptom severity, and the impact on quality of life. According to previous studies, a 4-point decrease was identified as the Minimal Clinically Important Difference.^[21, 22, 23]^

The secondary outcome included the change in the NIH-CPSI score from baseline to the 14-day treatment period.

Additionally, the rates of disappearance of individual core symptoms (including pelvic pain and lower urinary tract monosymptoms) and the overall effectiveness were recorded at the end of the 28-day treatment period.^[24]^ As SLA capsules are a traditional Chinese medicine (TCM) preparation, the influence of TCM symptoms needs to be considered; therefore, the resolution rate of these symptoms was additionally analyzed. TCM symptoms were referenced from the *Expert Consensus on Integrated Traditional Chinese and Western Medicine Diagnosis and Treatment of Chronic Prostatitis*, with core symptoms including urinary tract, radiating pain, sexual dysfunction, and systemic symptoms.^[25]^ Data were collected using a standardized TCM Symptom and Sign Checklist for Chronic Prostatitis, which contains 31 predefined symptom items (Supplementary Table 1). Each symptom item was evaluated in a binary manner (“present” or “absent”) and was assessed by investigators through patient inquiry at baseline and at each follow-up visit. All investigators involved in TCM syndrome assessment received standardized training on the use of the checklist prior to study initiation to ensure consistency of data collection across centers.

**Table 1:** NIH-CPSI scores of the patients before and after being treated with SLA capsules

Evaluation item	NIH-CPSI	Pain domain	Urinary domain	Symptoms severity	Life Quality
Baseline score	24.0 ± 5.56	8.7 ± 3.67	6.2 ± 1.99	4.0 ± 1.26	5.1 ± 0.94
Scores following a 14-day treatment	14.7 ± 5.93***	4.8 ± 3.67***	3.6 ± 1.91***	2.7 ± 1.39***	3.7 ± 1.14***
Scores following a 28-day treatment	8.9 ± 5.97***	2.7 ± 3.05***	2.2 ± 1.55***	1.7 ± 1.48***	2.3 ± 1.47***

***Statistically significant when compared with baseline values, *P* < 0.001. NIH-CPSI: National Institutes of Health Chronic Prostatitis Symptom Index; SLA: Shulie'an.

All outcome assessments were conducted at baseline, 14, and 28 days after treatment. This design allowed for intra-individual comparisons (pre-treatment *vs*. post-treatment) and subgroup analyses by severity,^[26]^ enabling a comprehensive evaluation of the therapeutic benefits of SLA capsules across the clinical spectrum of chronic prostatitis.

In addition, to evaluate the safety of the SLA capsules, all adverse events occurring throughout the trial were systematically assessed, including the emergence or exacerbation of clinically significant abnormal laboratory findings, the onset or aggravation of clinically significant signs or symptoms, and the development or progression of new diseases. The severity of adverse events and their causal relationship with the investigational product was determined by authorized investigators in accordance with the National Cancer Institute Common Terminology Criteria for Adverse Events, Version 5.0, and relevant guidelines.

### Statistical analysis

Analyses were conducted using SAS software (version 9.4). Continuous variables are summarized as means and standard deviations. Baseline demographic characteristics were analyzed descriptively, and protocol deviations were documented. The NIH-CPSI scores at baseline, day 14, and day 28 were tabulated. Within-group comparisons of score changes from baseline were performed using the paired *t*-test for data meeting assumptions of normality and homogeneity of variance or the Wilcoxon signed-rank test for skewed distributions or unequal variances. Categorical data are described using frequencies and percentages, with within-group comparisons made using the *chi-square* or Fisher’s exact test (when *chi-square* test assumptions were not met). Ordinal data were analyzed using the rank-sum or Cochran–Mantel–Haenszel test. Adverse events were coded using the Medical Dictionary for Regulatory Activities (MedDRA, version 25.0 or higher), with a focus on treatment-emergent adverse events. Statistical significance was set at a two-sided *P*-value < 0.05. For missing post-treatment efficacy data, the last observation carried forward method was applied, excluding participants whose pre- and post-treatment scores were inverted. Missing safety data were not imputed.

## Results

### Patient baseline characteristics

Three center institutions participated, and 100 male participants were recruited for the study. The baseline characteristics of the enrolled patients are presented in Supplementary Table 2. The mean age of the included patients was 33.5±7.70 years, and the mean BMI was 23.5±2.72 kg/m^2^. Patients were evaluated 14 and 28 days after treatment initiation. All enrolled patients completed the treatment and scheduled follow-up assessments, except for three patients who violated the protocol.

**Table 2:** Disappearance rate of individual NIH-CPSI symptoms after 28 days of treatment

Symptoms	Number of Cases (*n*)	Disappeared	Not Disappeared	Disappearance Rate*(%)
Pain symptoms	89	37	52	41.57
Incomplete-emptying sensation	97	28	69	28.87
Urgency within 2 h after urination	95	29	66	30.53

*The disappearance Rate was defined as the ratio of post-treatment symptom disappearance to baseline symptom disappearance. NIH-CPSI: National Institutes of Health Chronic Prostatitis Symptom Index.

### NIH-CPSI scores

**Figure 2:**
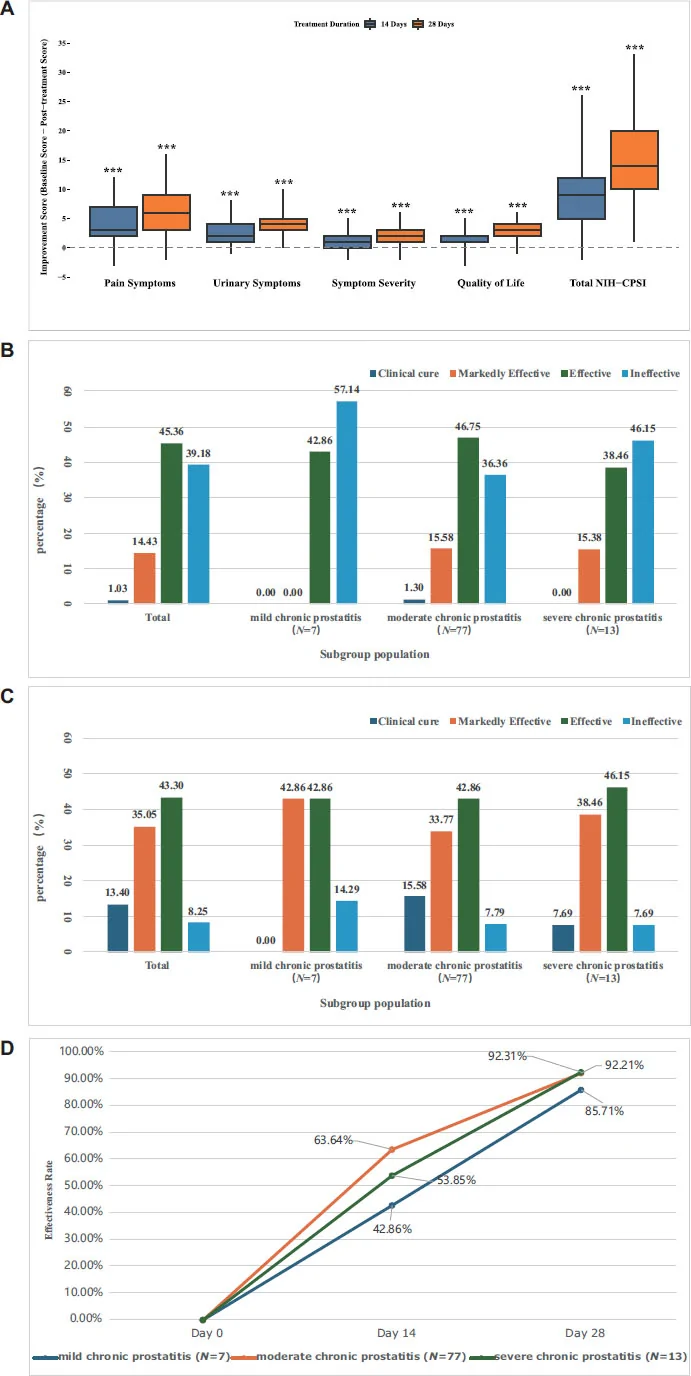
Improvement of NIH-CPSI scores during the study. (A) The change in NIH-CPSI score after treatment. Box plots were used to present the changes in the individual domain symptoms of the NIH-CPSI at Day 14 and Day 28 compared with the baseline, *** Statistically significant when compared to base line value, *P* < 0.001. (B) The efficacy of treatment after 14 days in different subgroups. A bar chart was used to illustrate the distribution of various types of clinical effectiveness among patients with different severity levels at Day 14. (C) The efficacy of treatment after 28 days in different subgroups. (D) The change in total effective rate after treatment in different subgroups. Total effective rate was calculated as the ratio of the sum of cured, markedly effective, and effective cases to the total number of participants. NIH-CPSI: National Institutes of Health Chronic Prostatitis Symptom Index.

After 14 and 28 days of SLA treatment, the NIH-CPSI total, pain, urinary domain, severity of symptoms, and quality of life scores significantly improved at follow-up evaluations compared with those at baseline (*P* < 0.001) (Table 1 and Figure 2A). The NIH-CPSI scores decreased 9.2±5.82 points after 14 days and 15.0±6.79 points after 28 days. After 28 days of treatment, the NIH-CPSI score reduced by more than 4 points compared with those at baseline in 95.88% of the participants. The overall response rate was 60.82% and 95.88% after 14 and 28 days of treatment, respectively. After 28 days of treatment, the disappearance rates of pain, incomplete emptying, and urgency within 2 h of urination were 41.57%, 28.87%, and 30.53%, respectively (Table 2).

We subsequently performed a subgroup analysis aimed at providing valuable insights into the therapeutic efficacy across patient groups with different severities of chronic prostatitis by analyzing outcomes within these subgroups. This analysis included a study population of chronic prostatitis patients stratified into three distinct severity levels based on the NIH-CPSI scores: 7 patients identified as having mild chronic prostatitis (NIH-CPSI score ≤ 14), 77 categorized as having moderate chronic prostatitis (NIH-CPSI score: 15–29), and 13 classified as having severe chronic prostatitis (NIH-CPSI score ≥ 30) (Supplementary Table 3). The results showed that after 14 and 28 days of treatment (Table 3 and Figure 2B-D), SLA capsules greatly improved the NIH-CPSI total, pain, urinary domain, severity of symptoms, and quality of life scores of patients with moderate and severe chronic prostatitis compared with those observed at baseline (*P* < 0.001). For patients with moderate disease, the results showed an overall efficiency of 63.64% after 14 days and 92.21% after 28 days of treatment. For patients with severe disease, the results showed an overall efficiency of 53.85% after 14 days and 92.31% after 28 days of treatment.

**Tables 3:** NIH-CPSI single symptom scores for different subgroups

Parameters of NIH-CPSI	Mild disease (*n* = 7)			Moderate disease (*n* = 77)			Severe disease (*n* = 13)		
	Baseline	Day 14	Day 28	Baseline	Day 14	Day 28	Baseline	Day 14	Day 28
Total score	13.1 ± 1.46	9.3 ± 2.43^†^	5.6 ± 1.90^†^	23.5 ± 3.83	14.3 ± 5.19^†^	8.5 ± 5.52^†^	32.5 ± 2.33	20.4 ± 7.38^†^	13.6 ± 7.63^†^
Pain domain score	1.4 ± 2.44	1.0 ± 1.73	0.4 ± 1.13	8.6 ± 2.86	4.6 ± 3.45^†^	2.6 ± 2.77^†^	12.8 ± 2.05	7.9 ± 3.38^†^	5.1 ± 3.97^†^
Urinary domain score	4.1 ± 1.35	3.1 ± 1.21	2.1 ± 1.21^*^	6.0 ± 1.83	3.4 ± 1.86^†^	2.1 ± 1.53^†^	8.3 ± 1.44	4.7 ± 2.25^†^	3.0 ± 1.68^†^
Severity of symptoms score	3.0 ± 1.53	2.1 ± 1.57	1.1 ± 1.07^†^	3.8 ± 1.12	2.7 ± 1.30^†^	1.6 ± 1.44^†^	5.5 ± 0.66	3.5 ± 1.56^†^	2.5 ± 1.71^†^
Quality of life score	4.6 ± 0.53	3.0 ± 0.58^*^	1.9 ± 0.69^†^	5.0 ± 0.97	3.6 ± 1.12^†^	2.2 ± 1.47^†^	5.9 ± 0.28	4.2 ± 1.30^†^	3.1 ± 1.61^†^

*Statistically significant when compared with baseline values, *P* < 0.05;

† Statistically significant when compared with baseline values, *P* < 0.01;

‡ Statistically significant when compared with baseline values, *P* < 0.001. NIH-CPSI: National Institutes of Health Chronic Prostatitis Symptom Index.

### Disappearance rate of TCM symptoms

**Figure 3:**
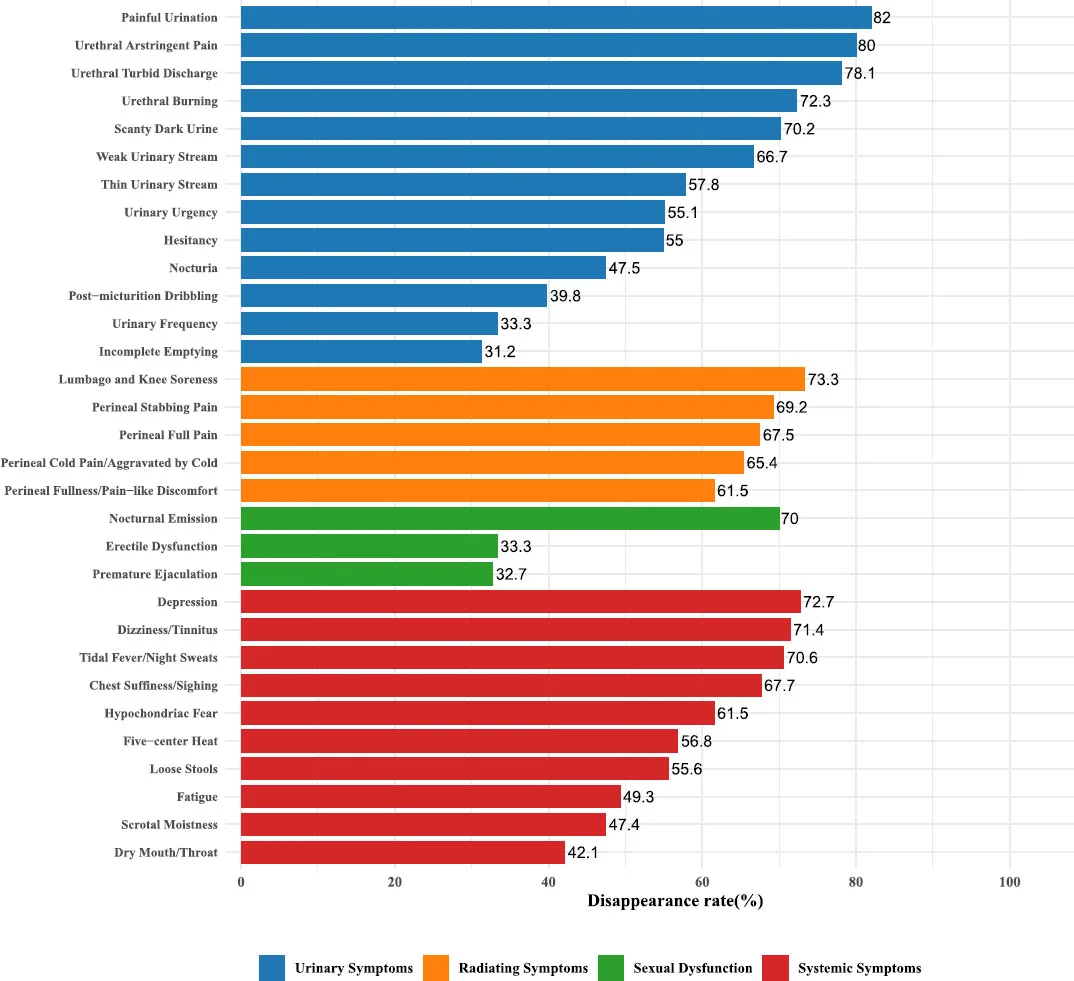
Disappearance rate of individual TCM symptoms after 4 weeks of treatment. TCM symptoms presented cover 4 categories, includes urinary tract symptoms, radiating pain symptoms, sexual dysfunction, and systemic symptoms, which involve 13, 5, 3, and 10 different key symptoms respectively. The proportion of each symptom was presented using R. TCM: traditional Chinese medicine.

Among the population enrolled in this trial, 100% presented with individual TCM urinary symptoms, with incomplete urination being the most common (99%), followed by frequent urination (96%), and post-void dribbling (91%). Symptoms of radiating pain were present in 72% of the patients, among which a sense of heaviness or dull pain in the perineum was the most common (53%), followed by soreness and weakness in the lower back and knees (46%) and hidden pain in the perineal region (4%). After 28 days of treatment (Figure 3), the disappearance rates of individual TCM symptoms were highest for painful urination (82%), followed by urethral burning or soreness (80%), turbid urethral discharge (78.13%), and soreness and weakness in the lower back and knees (73.33%).

### Adverse events and safety

The incidence of adverse events in this study was 11%, while that of adverse reactions “possibly related” to the drug was 6%—mainly manifesting as mild gastrointestinal symptoms, with no serious adverse events observed, suggesting that the SLA capsule is well tolerated and safe. All adverse events are detailed in Supplementary Table 4.

## Discussion

Chronic prostatitis represents a complex pain syndrome characterized by heterogeneous clinical manifestations, diagnostic challenges, and diverse treatment approaches.^[14,15]^ The condition typically presents with a range of symptoms beyond prostatic pain, including lower urinary tract symptoms, such as urinary frequency, post-micturition dribbling, and decreased urinary stream.^[8,27,28]^ TCM provides a holistic approach by simultaneously targeting inflammation, pelvic microcirculation, and neuropsychiatric pathways, which aligns with the multifactorial nature of chronic prostatitis.^[15,29]^

Our findings showed that the SLA capsules exhibit clinically meaningful efficacy in chronic prostatitis management, thereby validating and extending previous observations from multiple studies conducted among Chinese patients.^[12, 13, 14]^ Therapeutic effects were evident as early as day 14, with further progressive improvement observed through day 28, as evidenced by significant reductions across all NIH-CPSI domains (pain: -5.9 points; urinary symptoms: -4.0 points; symptom severity: -2.3 points; quality of life impact: -2.8 points). These results suggest the rapid onset of symptom relief and improvement in quality of life, a particularly valuable characteristic considering the chronic nature of this condition. Notably, while only a few mild cases were included in our study (*n* = 7), we observed an interesting efficacy gradient across the severity strata (85.71% in mild cases versus 92.21%–92.31% in moderate-to-severe cases). Subgroup analysis stratified by disease severity (Figure 2D) showed that patients with moderate and severe disease had significantly greater symptom improvement than did patients with mild disease at 14 days of treatment; at 28 days, their improvement diminished and converged to the level of patients with mild disease. Analysis of baseline symptom burden, clinical characteristics, and individual treatment response suggested that this phenomenon may relate to higher baseline symptom burden and natural temporal symptom fluctuation in patients with moderate and severe disease. Furthermore, adjusted baseline characteristic analyses confirmed that these efficacy differences were not due to baseline confounding factors. These data also demonstrate that SLA capsules are effective not only in patients with mild chronic prostatitis but also exert significant efficacy in those with moderate and severe disease—a population for which effective treatments are notably limited.

The analysis of the disappearance of individual TCM symptoms further supports our research findings. After 28 days of treatment, the highest disappearance rate was observed for painful urination, followed by burning or soreness in the urethra, turbid urethral discharge, soreness, and weakness in the lower back and knees. This finding indicates that the SLA capsules have clinical efficacy in improving pelvic-related symptoms and show a notable effect on core TCM syndromes, such as qi stagnation with blood stasis in the lower burner and damp-heat accumulation. From the perspective of the TCM theory,^[30, 31, 32]^ these symptoms are primarily associated with impaired bladder meridian qi, obstruction due to damp-heat, and kidney qi deficiency with failure to consolidate essence. Therefore, the mechanism of action of the SLA capsules may be closely related to their ability to clear damp heat and promote blood circulation to unblock the meridians; however, well-designed studies are required to confirm these findings.

The safety profile of the SLA capsules appears favorable, with an 11% overall adverse event rate and a 6% drug-related adverse reaction rate, as well as predominantly mild gastrointestinal symptoms. We hypothesized that these reactions may reflect the heat-clearing properties of the formula, a well-documented mechanism in TCM pharmacology.^[33, 34, 35]^ However, the study design has some limitations, primarily involving the open-label setting, tolerability assessment, and sample size considerations. As a standardized assessment of gastrointestinal function was not conducted at baseline, future trials should incorporate a comprehensive gastrointestinal function evaluation 1–2 weeks prior to treatment. Additionally, the sample sizes for subsequent studies should be calculated based on the results of this trial to more precisely distinguish the therapeutic effects of the drug from those attributable to patients' underlying disease status. Another key limitation regarding the control group design warrants attention: a parallel control group (*e.g*., a placebo group or a group receiving conventional Western medicine treatment) was not included in the initial study design. This limitation prevents the exclusion of interference from the placebo effect and the natural course of the disease; furthermore, the open-label design may introduce potential assessment bias in the evaluation of NIH-CPSI scores. Despite these limitations, the findings of this study still provide preliminary evidence for the clinical application of SLA capsules and confirm its potential value as a therapeutic option for chronic prostatitis. Future well-designed randomized, double-blind, controlled trials are necessary to further validate the conclusions of this study.

In summary, our findings reveal that SLA capsules have significant clinical efficacy and favorable safety in the treatment of chronic prostatitis. During the 28-day treatment period, the patients showed continuous improvement in clinical symptoms, with particularly notable relief of pain and significant reductions across all symptom scores. These results show that these capsules may be an effective therapeutic option for chronic prostatitis.

## Supplementary Material

Supplementary Material Details
