# Supplementary material for: Efficacy and safety of Shulie'an capsules in treating chronic prostatitis: A multicenter, prospective, open-label clinical trial
Source: J Transl Int Med. 2026 Aug 26;14(4):620–9. doi: 10.1515/jtim-2026-0078 (PMC13508833; doi:10.1515/jtim-2026-0078)
Supplement: Supplementary file 1 [file jtim-2026-0078_sm.pdf]

Supplementary materials

Appendix

Supplementary Table 1: TCM Symptom and Sign Checklist

Classification	Symptom	Absent	Present
Urinary tract symptoms	Urethral burning		
	Urethral soreness		
	Frequent urination		
	Urgency		
	Painful urination		
	Post-void dribbling		
	Short red urine		
	Turbid urethral discharge		
	Thin urine stream		
	Incomplete urination		
	Hesitancy		
	Weak urine stream		
	Nocturia		
Radiating pain symptoms	Perineal heaviness or distension, dull pain		
	Localized perineal stabbing pain		
	Perineal cold pain, worsens with cold exposure		
	Perineal vague pain		
	Weakness and soreness in the lower back and knees		
Sexual dysfunction	Premature ejaculation		
	Erectile dysfunction		
	Nocturnal emission		
Systemic symptoms	Scrotal dampness		

	Depressed mood
	Chest tightness with frequent sighing
	Hypochondriasis/health anxiety
	Fatigue and lack of energy
	Loose or watery stools
	Heat sensation in the palms, soles, and chest
	Tidal fever and night sweats
	Dizziness and tinnitus
	Dry mouth and throat

TCM: traditional Chinese medicine.

Supplementary Table 2: Baseline clinical characteristics of the patient cohort

**Characteristics**	**Parameters**
Age (years)	33.5 ± 7.70
Height (cm)	174.49 ± 5.36
Weight (kg)	71.65 ± 9.31
BMI (kg/m²)	23.5 ± 2.72
Body temperature (℃)	36.54 ± 0.29
Heart rate (beats/min)	74.6 ± 9.85
Respiratory rate (breaths/min)	17.6 ± 2.22
Systolic blood pressure (mmHg)	119.6 ± 9.83
Diastolic blood pressure (mmHg)	74.4 ± 8.13
NIH-CPSI scores	23.9 ± 5.51

BMI, body mass index; NIH-CPSI, National Institutes of Health Chronic Prostatitis Symptom Index

Supplementary Table 3: Efficacy of treatment for different subgroups of chronic prostatitis

Classification*	Total patients (*n* = 97)		Mild disease (*n* = 7)		Moderate disease (*n* = 77)		Severe disease (*n* = 13)	
Timelines	Day 14	Day 28	Day 14	Day 28	Day 14	Day 28	Day 14	Day 28
Clinical cure	1	13	0	0	1	12	0	1
Markedly effective	14	34	0	3	12	26	2	5
Effective	44	42	3	3	36	33	5	6
Ineffective	38	8	4	1	28	6	6	1

*Classification was based on the following criteria: (1) Clinical cure: score reduction ⩾ 95%; (2) Markedly effective: score reduction < 95% but ⩾ 60%; (3) Effective: score reduction < 60% but ⩾ 30%; (4) Ineffective: score reduction < 30% or no change. In addition, the data presented in this table represent the patient samples categorized according to these classifications.[35]

Supplementary Table 4: List of AEs during the trial

Drug number	Adverse event	Start date	End date	Severity	Relationship with investigational drug	Is it a serious adverse event?
1	Dyspepsia	2024-05-08	2024-05-09	Grade 1	Possibly related	No
12	Elevated urinary red blood cells	2024-07-24	2024-08-12	Grade 1	Possibly unrelated	No
14	Cold-like symptoms	2024-07-14	2024-07-18	Grade 1	Possibly unrelated	No
17	Constipation	2024-07-25	2024-07-28	Grade 1	Unrelated	No
17	Cold-like symptoms	2024-07-23	2024-07-25	Grade 1	Possibly unrelated	No
20	Abdominal pain	2024-08-07	2024-08-09	Grade 1	Possibly unrelated	No
20	Nausea	2024-08-07	2024-08-09	Grade 1	Possibly unrelated	No
27	Hematuria	2024-07-22	UK	Grade 1	Possibly related	No

36	Elevated urinary red blood cells	2024-08-06	2024-08-26	Grade 1	Possibly related	No
39	Elevated white blood cells	2024-08-14	2024-09-02	Grade 1	Possibly related	No
44	Diarrhea	2024-08-10	2024-08-11	Grade 1	Possibly related	No
54	Diarrhea	2024-08-23	2024-08-23	Grade 1	Possibly related	No
93	Sinus bradycardia	2024-08-17	2024-09-14	Grade 1	Unrelated	No

AEs, adverse events.
